# New Approaches, Findings, and Diagnostics in Medical and Surgical Retina

**DOI:** 10.1155/2015/193561

**Published:** 2015-01-29

**Authors:** Jerzy Nawrocki, Ron Adelman, Didier Ducournau

**Affiliations:** ^1^Ophthalmic Clinic Jasne Blonia, Lodz, Poland; ^2^Yale University Eye Center, New Haven, CT, USA; ^3^Clinique Sourdille, Nantes, France

The retina is a very innovative subspecialty of ophthalmology and ophthalmologists are continuing to play major roles in keeping this an exciting and dynamic field.

The amazing developments in OCT imaging, the use of smaller and smaller gauge instruments, and advances in cutting tools and pump systems all reflect the dedication of surgeons and the ophthalmic industry to improve patient outcomes through better diagnostics, machinery, software, and the technical aspects of surgical practice. The new investigations presented in this special issue focus on some of these current innovations. For example, the study by A. L. Sabater et al., on the effects of Brilliant Blue G-assisted internal limiting membrane peeling for idiopathic macular hole on the thickness of the macular retinal ganglion cell-inner plexiform layer, illustrates how new developments in OCT devices and software continue to provide new tools for ophthalmic examination. S. Koinzer et al. suggest a new OCT based classification system, which could enable comparison of differently created photocoagulation lesions across different studies and predictive estimation of clinical effects of photocoagulation. M. Pfister et al. have examined how we go about our surgical tasks at the detailed level of comparing reaction times for hand-powered versus foot-powered switch operations. The very latest development in OCT, Swept Source OCT, has been used by J. Michalewski et al. to measure choroidal thickness and volume.

Interestingly, some of the new studies presented in this issue show that older techniques remain as important today as ever. The electroretinogram can be brought into the 21st Century by using DWT scalograms according to a paper by M. Gauvin et al. New drug therapies have changed the way that many diseases are treated yet the EVRS present two of the largest studies ever conducted, into DME and RVO both of which highlight that despite the acknowledged benefits of anti-VEGF injections, vitrectomy with ILM peeling might provide the best visual outcomes for patients suffering from these diseases. D. Cohen et al. demonstrate the benefits of primary scleral buckling when dealing with posterior segment open-globe injuries.

This special issue brings you innovation in action: new applications for well-known techniques, modern imaging and technical systems, novel methods of acquiring and dealing with data, and complex statistical analysis. Retina specialists are seriously involved in constant research and development. Some of the papers presented here state the need for further investigation in order to develop their findings and peer-reviewed publications have a key role to play. They not only give us the opportunity to share our latest research but also provide a springboard to push that research ever onwards, leading to the development of new strategies to treat retinal diseases. I hope that you will enjoy the articles here and that they might encourage all of you to join in the many studies that are currently underway and to aim to publish your own material.

*Jerzy Nawrocki* *Ron Adelman* *Didier Ducournau*

